# Exploring the *In Vivo* Role of the Mitochondrial Calcium Uniporter in Brown Fat Bioenergetics

**DOI:** 10.1016/j.celrep.2019.04.013

**Published:** 2019-04-30

**Authors:** Daniel Flicker, Yasemin Sancak, Eran Mick, Olga Goldberger, Vamsi K. Mootha

**Affiliations:** 1 Howard Hughes Medical Institute and Department of Molecular Biology, Massachusetts General Hospital, Boston, MA 02114, USA; 2 Department of Systems Biology, Harvard Medical School, Boston, MA 02115, USA; 3 Broad Institute, Cambridge, MA 02141, USA; 4 Present address: Department of Pharmacology, University of Washington School of Medicine, Seattle, WA 98195, USA; 5 Lead Contact

## Abstract

The mitochondrial calcium uniporter has been proposed to coordinate the organelle’s energetics with calcium signaling. Uniporter current has previously been reported to be extremely high in brown adipose tissue (BAT), yet it remains unknown how the uniporter contributes to BAT physiology. Here, we report the generation and characterization of a mouse model lacking *Mcu*, the pore forming subunit of the uniporter, specifically in BAT (BAT-Mcu-KO). BAT-Mcu-KO mice lack uniporter-based calcium uptake in BAT mitochondria but exhibit unaffected cold tolerance, diet-induced obesity, and transcriptional response to cold in BAT. Unexpectedly, we found in wild-type animals that cold powerfully activates the ATF4-dependent integrated stress response (ISR) in BAT and up-regulates circulating FGF21 and GDF15, raising the hypothesis that the ISR partly underlies the pleiotropic effects of BAT on systemic metabolism. Our study demonstrates that the uniporter is largely dispensable for BAT thermogenesis and demonstrates activation of the ISR in BAT in response to cold.

## In Brief

Flicker et al. generate a mouse lacking mitochondrial calcium uniporter activity in brown fat. They show that the uniporter is dispensable for brown fat bioenergetics. Unexpectedly, they find that in wild type animals, cold stress induces ATF4 signaling in normal brown fat, suggesting a mechanism for cold-induced GDF15 and FGF21 elevation.

## Graphical Abstract

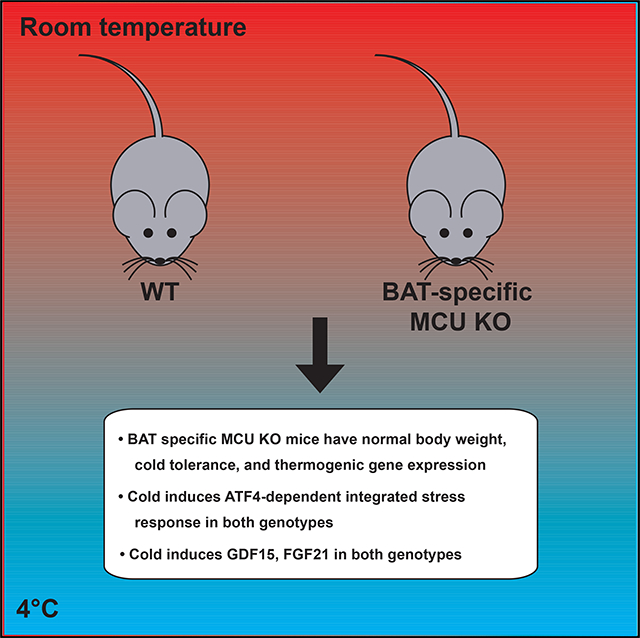

## INTRODUCTION

For decades, it has been known that calcium ions can enter mitochondria through a highly selective uniporter channel, driven by the electrochemical gradient across the inner mitochondrial membrane (IMM) (Carafoli and Lehninger, 1971; Deluca and Engstrom, 1961; Kirichok et al., 2004; Vasington and Murphy, 1962). Numerous studies have confirmed that calcium uptake in isolated mitochondria leads to transient dissipation of the membrane potential and sustained enhancement of NAD(P)H autofluorescence, oxygen consumption, and ATP phosphorylation (Territo et al., 2000). The effect on membrane potential is readily explained by the fact that the uniporter is electrophoretic, and hence calcium uptake leads to depolarization. A mechanism for the boost in NADH and oxidative phosphorylation was proposed as early as the 1970s, when it was noted that three matrix dehydrogenases are allosterically stimulated by calcium ions *in vitro* (for reviews, see Denton, 2009; Denton et al., 1996; McCormack et al., 1990).

Because energy-consuming events such as muscle contraction and neurotransmission are triggered by a release of calcium into the cytosol, it is plausible that these calcium boluses could enter mitochondria via the uniporter and trigger ATP production to match the increased cellular demand (Denton, 2009; Territo et al., 2000). This “feed-forward” model has historically been impossible to test, however, due to a lack of tools to selectively modulate the uniporter’s activity *in vivo.* The molecular identity of the uniporter began to be elucidated in 2010, enabling genetic disruption of its activity for the first time (Perocchi et al., 2010). At present, the mammalian uniporter is known to comprise five primary components, two of which are required to maintain a functional channel: the pore-forming subunit, MCU, and a small transmembrane binding partner, EMRE, both of which localize to the IMM (Baughman et al., 2011; Kovács-Bogdán et al., 2014; Sancak et al., 2013); MICU1 and MICU2, which are soluble subunits in the intermembrane space (IMS) that sense and gate the uniporter in the presence of subthreshold cytosolic calcium levels (Csordás et al., 2013; Kamer and Mootha, 2014; Kamer et al., 2017; Mallilankaraman et al., 2012); and the MCU homolog MCUb, which is thought to negatively regulate the uniporter’s conductance (Raffaello et al., 2013).

The molecular identification of this machinery has provided an unprecedented opportunity to delineate the role of the uniporter in cellular and organismal physiology. Several mouse models have since confirmed a role for the uniporter in supporting tissue bioenergetics, particularly when energy demand is acutely increased (Pan et al., 2013; Kwong et al., 2015; Luongo et al., 2015). In addition, a 2012 electrophysiology study by Fieni et al. (2012) demonstrated that the current density attributable to the uniporter is exceptionally high in mitoplasts isolated from skeletal muscle and brown adipose tissue (BAT) in comparison to liver, kidney, and heart. Skeletal muscle and BAT share a growing list of similarities (Seale et al., 2008; Stanford et al., 2013; Kim et al., 2013; Fisher et al., 2012), and both tissues respond to adrenergic signaling cues by acutely increasing energy consumption (Bachman et al., 2002; Lynch and Ryall, 2008). Because the uniporter has been shown to play a key bioenergetic role in skeletal muscle (Pan et al., 2013), we reasoned that it could serve a similarly important function in BAT; however, no studies to date have examined the role of the uniporter in this tissue.

BAT is a mammalian tissue specialized for metabolic inefficiency (Cannon and Nedergaard, 2004; Townsend and Tseng, 2014). It is densely packed with mitochondria and lipid droplets and is heavily innervated by sympathetic fibers that secrete norepinephrine (NE) in response to stimuli such as cold. NE acts on brown adipocytes primarily through the β_3_-adrenergic receptor, which signals through the cAMP-PKA pathway to liberate free fatty acids (FFA); these are oxidized to CO_2_ in the mitochondrial matrix but also activate uncoupling protein 1 (UCP1), a transporter that effectively permeabilizes the IMM to protons (Fedorenko et al., 2012; Wikstrom et al., 2014). NE therefore simultaneously stimulates rapid respiration and uncoupling in BAT, resulting in heat.

Early studies on isolated brown adipocytes found that intracellular calcium pools are mobilized by NE stimulation (Connolly et al., 1984), and subsequent imaging studies have repeatedly confirmed that NE induces a rise in cytosolic calcium in brown adipocytes (Chen et al., 2017; Leaver and Pappone, 2002; Lee et al., 1993; Nakagaki et al., 2005). However, it remains controversial whether this calcium is derived from mitochondria (Leaver and Pappone, 2002), and indeed whether NE stimulation causes a net rise or fall in matrix calcium levels (Hayato et al., 2011; Nakagaki et al., 2005). Although the physiological role of calcium signaling in BAT thermogenesis remains largely unexplored, it has recently been demonstrated that elevated cytosolic calcium can blunt heat production and thermogenic gene expression by repressing cAMP-PKA signaling (Chen et al., 2017; Nam et al., 2017). Elucidating the role of mitochondrial calcium handling in BAT may therefore provide significant insight into the regulation of thermogenesis.

In the present study, we generated and characterized a mouse model harboring BAT-specific loss of MCU (BAT-Mcu-KO). Despite ablated uniporter activity in this tissue, the animals did not exhibit an obvious phenotype: BAT bioenergetics were unimpaired as evidenced by intact cold-tolerance, susceptibility to diet-induced obesity, and thermogenic gene expression. Unexpectedly we found in wild-type animals that the integrated stress response (ISR) triggered by activating transcription factor 4 (ATF4) is strongly induced by cold in BAT.

## RESULTS

### Uniporter Expression Is Unremarkable in BAT

We first sought to determine whether the high uniporter current density reported in BAT mitochondria could be explained by high expression of MCU (Fieni et al., 2012). Prior large-scale studies found that MCU transcript levels are comparable between BAT, kidney, liver, and heart, and are approximately 2-fold higher in skeletal muscle (Su et al., 2002). Because these measurements do not correlate with the current densities reported in Fieni et al. (2012), we hypothesized that the uniporter components may vary more dramatically at the protein level across this panel of tissues. We therefore measured the abundance of MCU, EMRE, MICU1, and MICU2 in mitochondria isolated from each tissue (Figures S1A and S1B). We confirmed that these four components are highly enriched in skeletal muscle and depleted in heart. Surprisingly, however, the components were similarly expressed in BAT, kidney, and liver, at a level intermediate between that of skeletal muscle and heart. Thus, the current densities reported by Fieni et al. (2012) appear to correlate with uniporter protein levels in skeletal muscle, kidney, liver, and heart, but not in BAT.

### Generation of BAT-Specific MCU Knockout Mice

C57BL/6 mice harboring a full-body knockout of *Mcu* die prior to birth for unknown reasons (Murphy et al., 2014). Therefore, in order to examine the role of the uniporter in BAT, we deleted *Mcu* in a tissue-specific manner. We first generated a conditional *Mcu* allele in which the second exon is flanked by LoxP sites (Figure S2A). We then bred mice homozygous for this allele (*Mcu*^*fl/fl*^) with a transgenic line expressing Cre recombinase under control of the *Ucp1* promoter, which is highly specific to BAT (Kong et al., 2014). *Mcu*^*fl/fl*^ animals harboring the *Ucp1-Cre* transgene were born at the expected Mendelian ratio and were grossly indistinguishable from *Mcu*^*fl/fl*^ animals lacking the *Ucp1-Cre* transgene.

**Figure 1.**
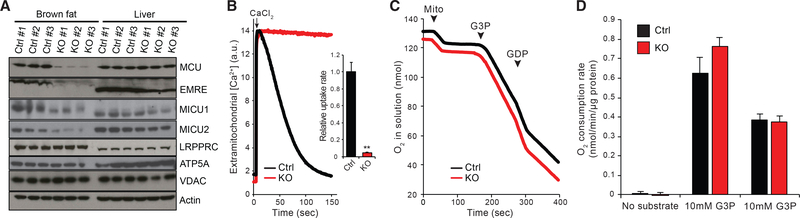
Selective Ablation of MCU in Brown Fat (A) Effect of brown fat-specific Cre recombinase expression on uniporter protein levels on the Mcu[fl/fl] background. (B) Left: representative Ca^2+^ uptake traces in isolated brown fat mitochondria energized with glycerol-3-phosphate (G3P) + rotenone in the presence of guanosine diphosphate (GDP). Right: quantification of Ca^2+^ uptake rates (n = 4). (C) Oxygen consumption measurements of isolated brown fat mitochondria in a well stirred cuvette at room temperature. Mitochondria, G3P, and GDP were added at indicated time points. (D) Quantification of oxygen consumption rates (n = 4). Results are reported as mean + SEM. Statistical significance is indicated as **p < 0.01 (Student’s t test).

We first confirmed that *Mcu*^*fl/fl*^*;Ucp1-Cre* (BAT-Mcu-KO) animals exhibited complete loss of MCU in BAT but not in liver, whereas *Mcu*^*fl/fl*^ (control) animals were unaffected (Figure 1A). EMRE levels were negligible in BAT-Mcu-KO BAT, consistent with reports that this protein is proteolytically degraded when unbound to MCU (Tsai et al., 2017), and MICU1 and MICU2 levels were reduced (Figure 1A). To ensure that uniporter activity was fully ablated, BAT mitochondria were isolated from BAT-Mcu-KO and control animals and energized with L-glycerol-3-phosphate (G3P) plus rotenone in the presence of GDP. As expected, control mitochondria exhibited a robust calcium uptake activity under these conditions, which was completely abrogated in BAT-Mcu-KO mitochondria (Figure 1B).

Our group previously demonstrated that OXPHOS is intact following MCU knockdown *in vivo* in liver, as demonstrated by intact State 3 to State 4 transitions in isolated mitochondria (Baughman et al., 2011). Consistently, mitochondria isolated from BAT-Mcu-KO and control BAT exhibited identical rates of oxygen consumption at baseline, in the presence of G3P (State 4u), and in the presence of both G3P and GDP (State 4) (Figures 1C and 1D).

### MCU Is Dispensable for BAT Bioenergetics

**Figure 2.**
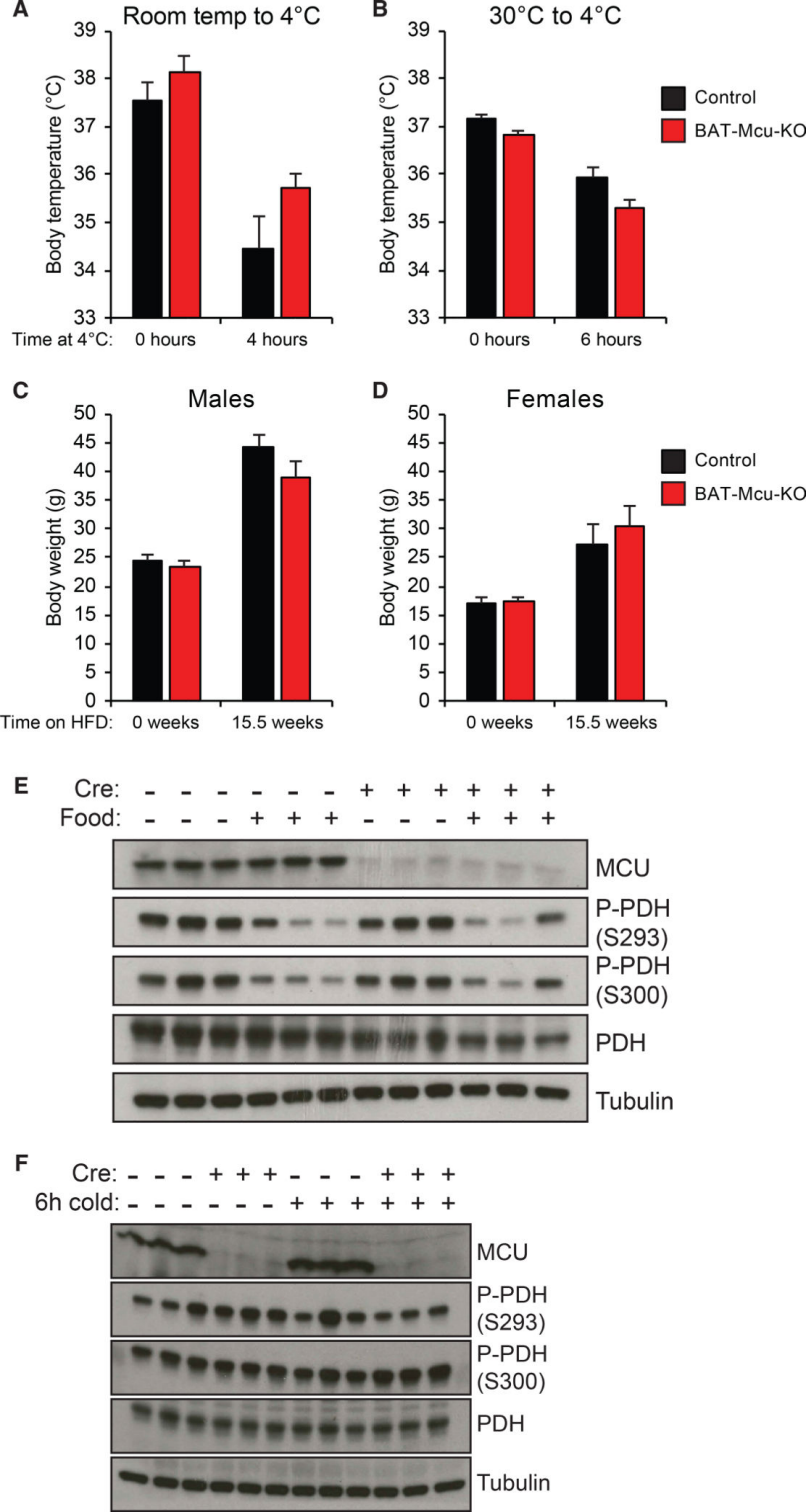
MCU Is Largely Dispensable for Brown Fat Bioenergetics (A) Core body temperature of mice transferred from room temperature to 4°C (n = 5–6 per group). Full body temperature data over time is presented in Figure S3A. (B) Core body temperature of mice transferred to 4°C following 1 week habituation to 30°C (n = 7 per group). Full body temperature data over time is presented in Figure S3B. (C and D) Body mass of male (C) and female (D) —Cre and +Cre animals fed high-fat diet. Full body mass data over time is presented in Figures S3C and S3D. (E) Brown fat pyruvate dehydrogenase (PDH) phosphorylation in —Cre or +Cre mice with or without *ad libitum* access to food at room temperature for 6 h. Animals were starved for 12 h overnight prior to the experiment. (F) Brown fat pyruvate dehydrogenase (PDH) phosphorylation in —Cre or +Cre mice fasted at room temperature or 4°C for 6 h. Results are reported as mean + SEM.

We next sought to determine the effect of MCU loss on BAT bioenergetics. Mice harboring lesions in mitochondrial bioenergetics in BAT are typically unable to maintain their body temperatures when acutely exposed to 4°C; thus, we speculated that BAT-specific MCU loss would confer increased sensitivity to cold stress (Bachman et al., 2002; Enerbäck et al., 1997; Lowell et al., 1993; Vergnes et al., 2011). Surprisingly, however, both BAT-Mcu-KO and control animals were able to defend their body temperatures to an equal extent when transferred from room temperature (RT) to 4°C (Figures 2A and S2B).

The response of BAT to a cold challenge is highly dependent on the temperature to which the animal has been habituated. Mice acclimated to 30°C conditions (thermoneutrality) exhibit an accumulation of unilocular lipid droplets and a mild reduction of mitochondria in interscapular BAT compared to mice housed at room temperature (22°C–26°C), and display a dramatic reduction in BAT oxygen consumption when administered a bolus of norepinephrine (NE) (Cannon and Nedergaard, 2011; Kozak, 2014). Conversely, even mice lacking UCP1 are able to defend their body temperatures at 4°C following 12 days of acclimation to mild cold (18°C) (Keipert et al., 2017). To test whether the role of the uniporter in nonshivering thermogenesis is dependent on the animals’ thermal prehistory, we habituated a cohort of mice to 30°C conditions for 1 week prior to administering a cold challenge. Both BAT-Mcu-KO and control animals were again able to defend their body temperatures to an equal extent (Figures 2B and S2C). We thus conclude that the uniporter is not required for body temperature maintenance via nonshivering thermogenesis.

Lesions in BAT thermogenesis have been shown to confer increased susceptibility to diet-induced obesity (Bachman et al., 2002; Feldmann et al., 2009; Lowell et al., 1993). To determine whether MCU loss constitutes such a lesion, we monitored body mass in BAT-Mcu-KO and control animals maintained on a high-fat diet (HFD) for 16 weeks. Consistent with our observations of cold tolerance, neither male nor female BAT-Mcu-KO animals exhibited a significant difference in body weight from age-matched control animals over the course of the experiment (Figures 2C, 2D, S2D, and S2E). Importantly we note that this study was conducted at room temperature, whereas prior studies have suggested that defects in diet-induced thermogenesis can generally only be observed at thermoneutrality (Enerbäck et al., 1997; Feldmann et al., 2009). It is therefore plausible that a genotype-dependent response to HFD might be evident at 30°C, although this is unlikely given that cold tolerance is unaffected by MCU.

The matrix enzyme pyruvate dehydrogenase phosphatase 1 (PDP1), which dephosphorylates and activates the pyruvate dehydrogenase (PDH) complex, has been shown to be allosterically activated by calcium *in vitro* (Denton et al., 1996; Huang et al., 1998). Consistently, CD1 total-body MCU knockout mice exhibit a decreased matrix calcium load and increased skeletal muscle PDH phosphorylation following a 16-h fast (Pan et al., 2013). In addition, skin fibroblasts isolated from patients with homozygous MICU1 loss exhibit an increased matrix calcium load and, as reported in a separate study, decreased PDH phosphorylation (Lewis-Smith et al., 2016; Logan et al., 2014). The uniporter has thus emerged as an important regulator of PDH phosphorylation under select conditions.

We speculated that MCU loss might impact PDH phosphorylation in BAT. To characterize the dynamics of PDH phosphorylation in BAT, mice were fasted overnight followed by 6 h of either additional fasting or *ad libitum* re-feeding. As has previously been demonstrated in skeletal muscle, re-feeding induced robust PDH dephosphorylation in BAT (Gudiksen and Pilegaard, 2017; Sugden et al., 2000). Interestingly, however, MCU loss did not impact refeeding-induced PDH dephosphorylation (Figure 2E).

Multiple studies have shown that adrenergic stimulation elicits a rise in cytosolic calcium in brown adipocytes (Chen et al., 2017; Leaver and Pappone, 2002). Mitochondrial calcium handling has been implicated in this process; however, it remains controversial if mitochondria subsequently take up the cytosolic calcium spike, or if they in fact contribute to it by releasing matrix calcium stores following UCP1-mediated depolarization (Connolly and Nedergaard, 1988; Lee et al., 1993; Nakagaki et al., 2005). We reasoned that according to either of these models, cold might impact PDH phosphorylation in BAT in an MCU-dependent manner. We therefore measured BAT PDH phosphorylation in BAT-Mcu-KO and control mice exposed to either RT or 4°C for 6 h. However, cold exposure did not substantially affect PDH phosphorylation in either genotype; MCU loss also had no effect on PDH phosphorylation at either temperature (Figure 2F).

Taken together, our observations suggest that uniporter function is largely dispensable for BAT bioenergetics, although we cannot exclude subtle effects that might be relevant in more natural environments.

### BAT Gene Expression Is Remodeled by Acute Cold Exposure Independently of MCU

**Figure 3.**
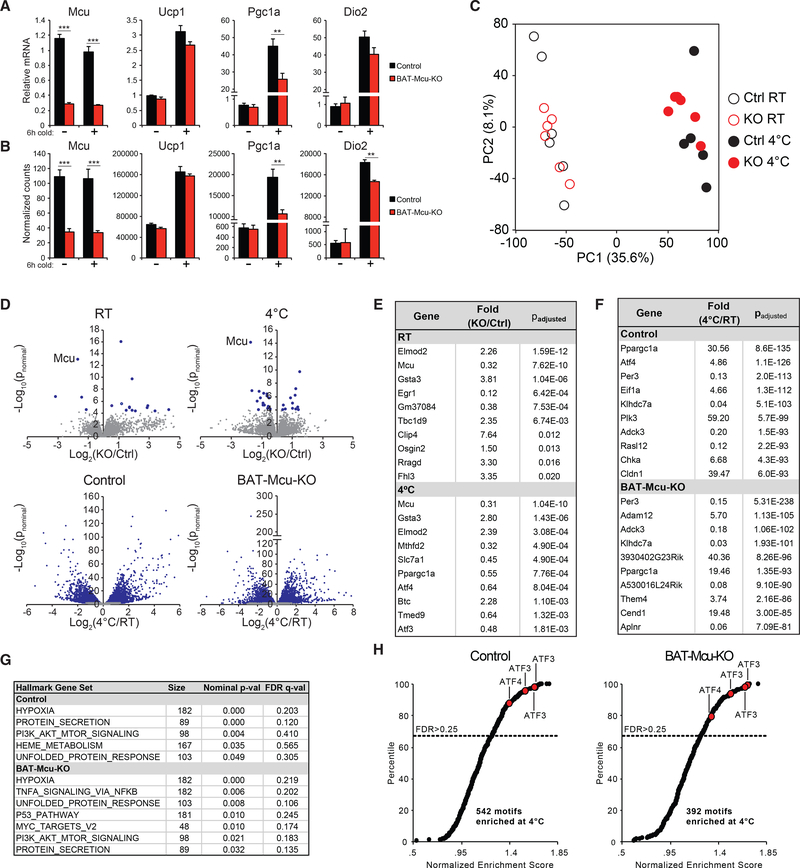
Transcriptional Response to Cold Exposure (A) qPCR of MCU and cold-induced transcripts in brown fat (n = 5–6 mice per group). (B) Normalized counts of transcripts in (A) measured by RNA-seq. (C) Principal component analysis of gene expression data. (D) Volcano plots comparing —Cre with +Cre gene expression at two temperatures, and comparing RT with 4°C gene expression in both genotypes (blue points denote transcripts achieving an adjusted p value < 0.05). (E) Top 10 genes enriched by genotype at either RT or 4°C (genes ranked by adjusted p value). (F) Top 10 genes enriched by temperature in either —Cre or +Cre mice. (G) MSigDB hallmark genesets enriched at 4°C in —Cre or +Cre mice. (H) DNA motifs enriched at 4°C in —Cre or +Cre mice. Red points indicate motifs annotated as ATF3 or ATF4 targets. Genes were rank ordered using the Student’s t test metric. Samples were permuted 1,000 times to evaluate significance. Results are reported as mean + SEM. Statistical significance is indicated as *p < 0.05, **p < 0.01, ***p < 0.001 (Student’s t test).

Because we were unable to detect a strong *in vivo* phenotype resulting from MCU loss in BAT, we next aimed to systematically quantify genome-wide gene expression in BAT in control and BAT-Mcu-KO animals. Prior studies of MCU knockout in skeletal muscle and heart revealed that, while no phenotype was evident at baseline, both tissues exhibited a blunted bioenergetic response to physiological stressors (exercise and adrenergic stimulation, respectively) (Kwong et al., 2015; Luongo et al., 2015; Pan et al., 2013). We chose cold as the relevant stressor in the present study and performed global transcriptional profiling on BAT from control and BAT-Mcu-KO animals exposed to RT or 4°C for 6 h. As a positive control for cold exposure, we confirmed that UCP1, PGC1A, and DIO2 were strongly upregulated in 4°C exposed mice in a genotype-independent manner (Ye et al., 2013); in contrast, MCU was strongly reduced in BAT-Mcu-KO animals but was transcriptionally unaffected by temperature (Figures 3A and 3B).

We observed relatively few significant transcriptional changes resulting from MCU loss at either RT or 4°C (Figures 3D and 3E). Besides *Mcu,* we identified three genes that were differentially expressed by genotype at both RT and 4°C: ELMOD2, TBC1D9, and GSTA3, all of which are upregulated in BAT-Mcu-KO BAT (STAR Methods). *Elmod2* and *Tbc1d9* are located in close proximity to *Ucp1* in the mouse genome and are likely overexpressed due to their presence in the UCP1-Cre transgene. *Gsta3* encodes a cytosolic enzyme that participates in the biosynthesis of steroid hormones (Johansson and Mannervik, 2001; Raffalli-Mathieu et al., 2008). Its significance in brown fat and its connection to mitochondrial calcium homeostasis are unclear.

In stark contrast to the mild effect of genotype, we found that over 15% of detected genes were significantly up or downregulated by at least 2-fold between RT and 4°C (Figures 3D and 3F). Principal component analysis (PCA) yielded robust clustering of samples by temperature along the first principal component axis, confirming just how pervasive and strong the thermogenic gene expression program is (Figure 3C). *Pgc1a, Dio2,* and *Ucp1* all scored within the top 100 genes in the loading of principal component 1 (STAR Methods).

When we sorted the genes enriched at 4°C in order of significance, we found that the transcription factor ATF4 was among the top 10 most significantly enriched genes in control and Mcu-KO BAT (Figure 3F) (Han et al., 2013). ATF4 is a highly studied molecule that orchestrates the integrated stress response (ISR), a gene expression program activated in response to a wide variety of perturbations including endoplasmic reticulum (ER) unfolded protein stress, amino acid or glucose deprivation, and severe hypoxia (Han et al., 2013; Harding et al., 2003; Pakos-Zebrucka et al., 2016). To investigate whether the canonical ISR was induced by cold in our experiment, we performed gene set enrichment analysis (GSEA) to identify hallmark gene sets and promoter motifs enriched at 4°C compared to RT (Mootha et al., 2003; Subramanian et al., 2005). The hypoxia and unfolded protein response hallmark gene sets, both of which contain reported ATF4 targets (Bao et al., 2016; Han et al., 2013), were significantly enriched in both control and BAT-Mcu-KO mice (Figure 3G), as were all four motifs annotated as binding to ATF4 or its downstream target ATF3 (Figure 3H).

### Cold Induces the ATF4-Associated Integrated Stress Response in BAT

**Figure 4.**
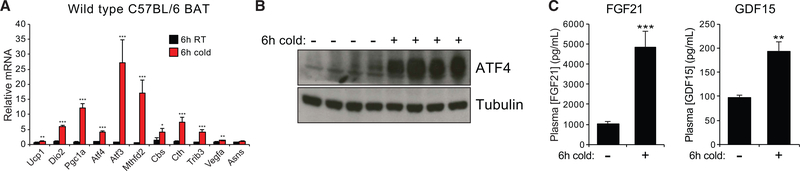
Cold Challenge Activates the Integrated Stress Response in Brown Fat (A) qPCR of transcripts corresponding to the thermogenic gene expression or the integrated stress response (ISR) in wild type mice housed for 6 h at RT or 4°C (n = 6 mice per group). (B) BAT ATF4 protein levels in fasting mice housed for 6 h at RT or 4°C. (C) Circulating levels of FGF21 and GDF15 in mice starved for 6 h at RT or 4°C (n = 7 mice per group). Results are reported as mean + SEM. Statistical significance is indicated as *p < 0.05, **p < 0.01, ***p < 0.001 (Student’s t test).

In order to validate that the canonical ISR is operative in BAT at 4°C, we first confirmed that several ATF4 target genes were robustly upregulated by cold in an independent cohort of mice (Figure 4A). We additionally found that treatment of differentiated immortalized brown adipocytes with NE induced both uncoupled respiration and upregulation of ATF3 within 4 hours, suggesting that adrenergic stimuli may induce the ISR in a cell-autonomous manner (Figures S3A–S3D).

ATF4 is acutely regulated at the translational level by the initiation factor eIF2α, which is itself activated upon phosphorylation by one of four protein kinases (for review, see Pakos-Zebrucka et al., 2016). Although the phosphorylated form of eIF2α broadly represses translation initiation, it also promotes skipping of a uORF in the ATF4 mRNA, leading to increased translation of the main coding region and rapid accumulation of ATF4 protein (Dey et al., 2010; Vattem and Wek, 2004). Consistently, we found that cold exposure led to accumulation of ATF4 at the protein level (Figure 4B). Interestingly, ATF4 accumulation was suppressed when mice were given *ad libitum* access to food over the course of the cold exposure period, regardless of whether animals were fasted beforehand (Figures S4A and S4B).

What purpose does cold induction of the ISR serve in BAT? To address this question, we noted that two genes known to be induced as part of the ISR are the cytokines FGF21 and GDF15 (Jousse et al., 2007; Khan et al., 2017; Kim et al., 2013). Both cytokines have been demonstrated to promote glucose tolerance, insulin sensitivity, resistance to diet-induced obesity, and upregulated expression of lipolysis and beta-oxidation genes in white and beige fat (Chrysovergis et al., 2014; Chung et al., 2017; Kharitonenkov et al., 2005; Kim et al., 2013; Fisher et al., 2012; Xiong et al., 2017). Furthermore, cold exposure has been shown to induce FGF21 transcription in BAT after 4–6 h and a 2-fold rise in circulating FGF21 levels after 24 h, raising the possibility that FGF21 may mediate the pleiotropic effects of BAT on systemic metabolism (Chartoumpekis et al., 2011; Hondares et al., 2011). We reasoned that, if ISR activation in BAT serves such a purpose, then both circulating FGF21 and GDF15 should be raised after 6 h of cold exposure. Indeed, we found that circulating FGF21 and GDF15 levels increased by nearly 5-fold and 2-fold, respectively (Figure 4C). To our knowledge, cold-induction of circulating GDF15 has not previously been reported. In order to determine whether any tissues besides BAT secrete significant quantities of FGF21 and GDF15 during cold exposure, we measured transcript levels of these and other ISR target genes in a panel of tissues known to be physiological sources of either hormone (Estall et al., 2009; Kempf et al., 2006; Khan et al., 2017) (Figure S4C). Among the tissues examined, BAT demonstrated by far the strongest transcriptional upregulation of FGF21 and GDF15. While we cannot rule out the possibility that other tissues contribute to the circulating FGF21 and GDF15 pools during cold exposure, these results suggest that the increase in both hormones may be primarily attributable to their production in BAT. Collectively, our observations indicate that the ISR may support many of the endocrine functions of BAT implicated in modulating systemic metabolism; furthermore, in addition to FGF21, thermogenic BAT may serve as an important physiological source of GDF15.

## DISCUSSION

The current study indicates that the mitochondrial calcium uniporter is largely dispensable for BAT bioenergetics: the mouse model we used completely lacks uniporter activity in BAT, but displays no obvious abnormalities in cold tolerance, diet-induced obesity, BAT PDH phosphorylation, or BAT transcriptome-wide gene expression at baseline or in response to acute cold challenge. Although not the goal of the current study, examining the global gene expression response to cold led us to identify a robust induction of the ISR in BAT in wild type animals. While determining the full functionality of the ISR in thermogenic BAT is beyond the scope of this study, we speculate that it may support the endocrine role of BAT by strongly inducing secretion of cytokines such as FGF21 and GDF15.

The uniporter has already been demonstrated to support exercise tolerance in skeletal muscle and sympathetic contractility stimulation in heart, both of which are highly energy-demanding processes linked to adrenergic signaling (Kwong et al., 2015; Luongo et al., 2015; Pan et al., 2013). Given that non-shivering thermogenesis is another such process, combined with the fact that a previous study identified the uniporter current to be extremely high in BAT (Fieni et al., 2012), we were surprised to discover the minimal role of the uniporter in BAT bioenergetics.

The study of various MCU knockout models has implicated a crucial role for genetic background in unmasking uniporter-associated phenotypes. The most dramatic example is, of course, the embryonic lethality of inbred full-body MCU knockout mice compared with the mild phenotype of the CD1 MCU knockout model (Murphy et al., 2014). Because we observed that MCU knockout results in complete ablation of uniporter activity in BAT, it is clear that the function of MCU is non-essential in this tissue on the C57BL/6 background.

While the uniporter is thought to mediate the only rapid mitochondrial calcium uptake pathway, it is certainly not the only means by which mitochondrial calcium influx can occur. For example, a mechanism that can theoretically sustain mitochondrial calcium homeostasis in the absence of uniporter function is reversal of the IMM sodium-calcium exchanger, NCLX, which ordinarily serves as the primary mitochondrial calcium efflux pathway (De Marchi et al., 2014; Palty et al., 2010). NCLX reversal may therefore play a greater role in mitochondrial calcium homeostasis than the uniporter in thermogenic BAT.

It is noteworthy that PDH is acted upon by two phosphatases: PDP1 and PDP2, the latter of which is calcium-insensitive (Huang et al., 1998). Previous studies (Su et al., 2002) and our own RNA sequencing (RNA-seq) data indicate that in BAT, PDP2 is over 10-fold more abundant than PDP1 at RT, and over 20-fold more abundant at 4°C (STAR Methods). It is therefore reasonable to expect that calcium exerts relatively little control over the PDH phosphorylation state in BAT, consistently with our results (Figures 2E and 2F).

Given that BAT mitochondria contain substantially less MCU and EMRE protein than skeletal muscle mitochondria (Figures S1A and S1B), it is unclear why Fieni et al. (2012) observed that mitoplasts from these two tissues harbor equally high current densities. Notably, the BAT mitoplasts used for patch clamp experiments in this study were isolated from mice lacking UCP1, suggesting that the high uniporter current density in BAT may be specific to this genotype; indeed, a recent quantitative proteomics study demonstrated that MCU, EMRE, MICU1, and MICU2 are all upregulated by ~2-fold in UCP1 knockout BAT (Kazak et al., 2017).

Importantly, an additional use for the BAT-Mcu-KO model that we have not yet explored is to examine the role of MCU in beige fat, a UCP1-expressing cell type dispersed primarily throughout the inguinal fat pad (Wu et al., 2012). Unlike interscapular BAT, the abundance of beige fat is relatively low at room temperature and is strongly increased following multi-day cold exposure (Young et al., 1984) or chronic, repeated administration of a β_3_-adrenergic agonist (Bertholet et al., 2017). Recent studies have identified multiple UCP1-independent pathways by which beige, but not brown, adipocytes can generate heat (Bertholet et al., 2017; Kazak et al., 2015), including futile cycling of calcium across the endoplasmic reticulum membrane (Ikeda et al., 2017); it is thus conceivable that MCU plays a substantive role in this tissue’s bioenergetics despite its dispensability in BAT.

An unexpected finding from our work is that cold powerfully induces the ATF4-dependent ISR in BAT. Relatively few studies have addressed the dynamics and functional significance of the ISR in this tissue (Bettaieb et al., 2012; Liu et al., 2017; Mamady and Storey, 2008; Sekine et al., 2016; Seo et al., 2009; Wang et al., 2010, 2013), and the interplay between the ISR and the thermogenic gene program is poorly understood. One recent study showed that mice lacking the mitochondrial Ser/Thr-specific protein phosphatase PGAM5 have increased levels of phospho-eIF2α and FGF21 in BAT following a 12-h fast and 3–6 h cold exposure; cold-induction of FGF21 mRNA was also blunted by treatment with ISRIB, which blocks the downstream effects of eIF2α phosphorylation (Anders and Huber, 2010; Sekine et al., 2016). Interestingly, while fasting plus cold also induced FGF21 transcription in wild type BAT, only an ~2-fold change in FGF21 mRNA was observed relative to fasted mice at RT. Our study validates and extends this finding by showing that acute cold exposure induces ATF4 protein accumulation, reproducibly engages the full ISR at a genome-wide level, and leads to a rapid and substantial increase of FGF21 and GDF15 in the circulation. We have also shown that fasting animals prior to cold exposure is not necessary for induction of the ISR in BAT, although feeding during cold exposure suppresses ATF4 accumulation; this suggests that the ISR is likely to be operative in BAT under normal physiological circumstances, and not just in the controlled setting of a 12-h fast. Furthermore, in our hands, a 6-h cold exposure increases FGF21 mRNA levels in BAT by up to 50-fold (STAR Methods).

Three other studies have directly explored the connection between ATF4 and BAT thermogenesis, all utilizing a full-body *Atf4* knockout mouse model (Seo et al., 2009; Wang et al., 2010, 2013). Surprisingly, these mice are smaller and have a lower body fat percentage than wild type mice; they are more insulin-sensitive and have higher resting energy expenditure both on chow and high-fat diets; they better maintain core body temperature during a 3-h cold challenge; and they exhibit mild upregulation of UCP1, PGC1A, select lipolysis genes, and select b-oxidation genes in BAT. One of these studies examined gene expression in wild type mice in response to a 7-h cold challenge and surprisingly found ATF4 levels to slightly decrease at 4°C (Wang et al., 2013). The same study proposed a model in which ATF4 displaces the cAMP-responsive transcription factor CREB from the PGC1A promoter, thereby repressing cold-induced PGC1A upregulation. Our results show that ISR activation in response to cold is highly dependent on the presence or absence of food (Figures S4A and S4B); this or other environmental factors may explain the discrepancy in ATF4 dynamics between the aforementioned study and ours.

Further work will be required to fully understand the interplay between the ISR, ATF4, FGF21, GDF15, and the thermogenic gene program in BAT. Additional studies will also be required to precisely delineate the conditions under which the ISR is activated by cold in BAT, particularly given that this effect is highly dependent on feeding status (Figures S4A and S4B). Notably, ATF4 signaling is also upregulated in multiple cellular and animal models of mitochondrial dysfunction (Bao et al., 2016; Quirós et al., 2017; Tyynismaa et al., 2010), including forced uncoupling via FCCP treatment (Quirós et al., 2017) or ectopic UCP1 expression (Keipert et al., 2014; Ost et al., 2015). Consistently, both FGF21 and GDF15 have emerged as promising blood biomarkers for human mitochondrial disorders (Davis et al., 2013; Lehtonen et al., 2016; Montero et al., 2016; Yatsuga et al., 2015). Future efforts to decipher the mechanism and physiological consequences of cold-induced ATF4 signaling in BAT may therefore help elucidate the role of the ISR in mitochondrial disease.

## STAR+METHODS

### CONTACT FOR REAGENT AND RESOURCE SHARING

Further information and requests for resources and reagents should be directed to and will be fulfilled by the Lead Contact, V.K.M. (vamsi@hms.harvard.edu).

### EXPERIMENTAL MODEL AND SUBJECT DETAILS

#### Mice

Mouse experiments were performed according to procedures approved by the Massachusetts General Hospital Institutional Animal Care and Use Committee. All mice were housed in groups of up to 5 animals in a room temperature facility with 12-hour light and dark cycles, and were provided access to a standard rodent chow diet. B6.FVB-Tg(Ucp1-cre)1Evdr/J (UCP1-Cre) mice described in Kong et al. (2014) were obtained from The Jackson Laboratory. All experimental animals were obtained by breeding MCU[fl/fl] mice with MCU[fl/fl];UCP1-Cre mice to yield a 1:1 ratio of progeny harboring zero or one copy of the UCP1-Cre transgene. Each experiment was performed using sex- and age-matched mice; animals used for cold tolerance and RNA-seq experiments were males at 8–12 weeks of age.

### METHOD DETAILS

#### Generation of MCU^fl/fl^ Mouse Line

BAC RP23–371B1 was purchased from BacPac Resources and was modified to flank exon 2 of *Mcu* with LoxP sites using recombineering as described (Sharan et al., 2009). ES cells from a mixed genetic background (129/BL6) were injected with the modified BAC (Figure S2) in the Beth Israel Deaconess Medical Center Transgenic Core Facility. Neomycin resistant ES cells were further screened with PCR and Southern blotting. ES cells with correct targeting were used to generate MCU^*fl/fl*^ mice. MCU^*fl/fl*^ mice were then crossed with wild-type C57BL/6J until ~95% background homogeneity was achieved by single nucleotide polymorphism (SNP) analysis (The Jackson Laboratory).

#### Isolation of Mouse Tissue Mitochondria

Crude mitochondria isolation was performed essentially as described by Fieni et al. (2012), with all steps performed at 0–4°C and BAT harvested from the interscapular region. Briefly, mice were sacrificed by CO_2_ asphyxiation followed by cervical dislocation. Tissues were dissected immediately, rinsed briefly in ice-cold PBS, and immersed in 10mL ice-cold isolation buffer (250mM sucrose, 10mM HEPES, 1mM EGTA, pH 7.25 with KOH). Tissues were then finely minced and homogenized with six slow strokes of a Potter-Elvehjem homogenizer rotating at 280 (liver) or 600 (brown adipose tissue, skeletal muscle, kidney, heart) rpm. To increase mitochondrial yield, the homogenate was centrifuged at 700*g* for 5 mins, and the resulting nuclear/unbroken cell pellet was resuspended in the same supernatant and homogenized again as above. The homogenate was then centrifuged at 700*g* for 10 mins, and the supernatant was collected and centrifuged at 8,500*g* for 10 mins. The resulting mitochondrial pellet was rinsed with 1mL isolation buffer and resuspended in 5mL isolation buffer. The 700*g* and 8,500*g* centrifugation steps were then repeated, and the resulting mitochondrial pellet was resuspended in approx. 200 μL isolation buffer. Mitochondrial protein content was measured by lysing a small sample in RIPA buffer and performing a Bradford assay.

#### Cold Exposure and Body Temperature Measurements

Age and sex-matched animals were individually housed at 4°C for up to six hours in pre-cooled cages without bedding, with *ad libitum* access to pre-cooled water. Animals at 4°C did not have access to food unless otherwise indicated; if food was provided, it was pre-cooled to 4°C overnight. Body temperature was measured rectally at indicated time points using a Physitemp BAT-12 thermometer outfitted with a RET-3 probe. All animals used for cold tolerance and RNA-seq experiments were males at 8–12 weeks of age.

#### High Fat Diet

Age and sex-matched animals at 6–10 weeks of age were housed with 1–2 animals per cage, and standard chow was replaced with a diet containing 60% kcal from fat (Research Diets formula D12492). Animal weights and average food intake were measured by hand twice weekly at approx. the same time of day.

#### BAT Mitochondrial Calcium Uptake Measurements

BAT mitochondrial calcium uptake measurements were performed according to Al-Shaikhaly et al. (1979). 30 mg of crude BAT mitochondria in isolation buffer was centrifuged at 8,500g for 10 mins at 4°C, and the resulting pellet was resuspended in 150 μL calcium uptake buffer (125mM sucrose, 20mM Tris-HCl, pH 7.2 with Tris base) supplemented with 2 μM rotenone, 1mM GDP, 0.1% bovine serum albumin (fatty acid free), and 1 μM membrane impermeable Oregon Green BAPTA-6F. Immediately prior to calcium uptake measurement, the medium was supplemented with 5mM L-glycerol-3-phosphate and mixed by gentle agitation. Fluorescence was monitored with a PerkinElmer Envision plate reader before and after injection of 50 μM CaCl_2_ using FITC filter sets (Ex485/Em535), with a 0.5 s measuring interval. Calcium uptake rates were calculated using the linear fit of uptake curves between 40–60 s.

#### BAT Mitochondrial Oxygen Consumption Measurements

30 μg of BAT crude mitochondria in isolation buffer was centrifuged at 8,500*g* for 10 mins at 4°C, and the resulting pellet was resuspended in calcium uptake buffer (125mM sucrose, 20mM Tris-HCl, pH 7.2 with Tris base) supplemented with 0.1% bovine serum albumin (fatty acid free) and 0.5uM tetramethylrhodamine methyl ester. Mitochondria were then added to a well-stirred cuvette at 25°C to reach 500 μL total volume. At indicated time points 1mM L-glycerol-3-phosphate and 1mM GDP were added. O2 consumption and membrane potential were measured simultaneously using a custom spectrophotometer outfitted with an Ocean Optics benchtop NeoFox-GT phase fluorimeter, as previously described (Gohil et al., 2013).

#### Western Blotting

Animals were sacrificed by CO_2_ asphyxiation followed by cervical dislocation. Tissues were immediately harvested and snap frozen in liquid N_2_. For preparation of protein lysates, 1 BAT depot (or equivalent volume of another tissue) was immersed in approx. 300 μL ice-cold RIPA buffer supplemented with either complete EDTA-free protease inhibitor cocktail or Protease/Phosphatase Inhibitor Cocktail. The tissue was then lysed with two 5mm stainless steel beads using a QIAGEN TissueLyser for 2 mins at 25 Hz. The resulting homogenate was centrifuged for 10 mins at maximum speed at 4°C, and the supernatant was centrifuged a second time to remove residual insoluble material. Protein content of the resulting clarified lysate was determined using a Bradford assay. Appropriate volumes of lysate were boiled for 5 mins in the presence of SDS sample buffer, resolved on Tris-Glycine SDS-PAGE gels, and transferred to PVDF membranes for western blotting. All antibodies used are listed in the key resources table.

#### RNA isolation from BAT

Animals were sacrificed by CO_2_ asphyxiation followed by cervical dislocation, and tissues were immediately harvested and snap frozen in liquid N_2_. Frozen BAT samples were homogenized in 1mL Qiazol per 100mg tissue using the QIAGEN TissueRuptor II. The homogenate was mixed thoroughly with chloroform (1:5 chloroform:homogenate), incubated for 3 mins at room temperature, and centrifuged at 12,000*g* for 15 mins at 4°C, and 100 μL of the resulting aqueous phase was added to 350 μL QIAGEN buffer RLT plus 250 μL of 100% ethanol. The resulting mixture was transferred to a column from the QIAGEN RNeasy Mini Kit, and RNA was purified according to the manufacturer’s protocol.

#### Quantitative Real-Time PCR

RNA was reverse transcribed using the SuperScript III First-Strand Synthesis SuperMix Kit according to the manufacturer’s protocol. Quantitative real-time PCR (qPCR) was performed using TaqMan assays; all probe IDs are listed in the key resources table. Relative gene expression was calculated as 2^−ΔΔCT^, where HPRT was used as the housekeeping gene for normalization.

#### RNA-seq

RNA-sequencing libraries were prepared by the Broad Technology Labs at the Broad Institute based on the Smart-seq2 protocol (Picelli et al., 2014) and sequenced on an Illumina NextSeq 500 instrument to generate 2×25bp paired-end reads. The reads were aligned to the mouse genome (mm10, with gencode M7 annotations) using STAR 2.4.0j (default parameters) (Dobin et al., 2013). Counts of reads uniquely mapping within exonic regions of annotated genes (irrespective of strand) were collated using HTSeq (Anders et al., 2015). 14,651 genes with at least 8 reads mapping to them in at least 6 of the samples were retained for differential expression analysis. This was performed in R using DESeq2 (Love et al., 2014) and the design formula ~Genotype + Temperature + Genotype:Temperature. Where gene expression levels are reported, they represent normalized read counts following application of the estimateSizeFactors function, which implements the median ratio method. Where log2 fold-changes and p values are reported, they represent the result of the Wald test. P values are adjusted with the method of Benjamini-Hochberg. Further data analysis based on the normalized read counts was performed in MATLAB. Gene Set Enrichment Analysis (GSEA) was performed as described in Mootha et al. (2003) and Subramanian et al. (2005). All sequencing results have been deposited in NCBI GEO with the accession number GSE119964.

#### Cytokine assays

Mice were sacrificed by CO_2_ asphyxiation and blood was immediately drawn from the inferior vena cava. To obtain plasma, blood was incubated in EDTA-treated tubes and centrifuged at 14,000rpm for 10 mins at 4°C. FGF21 and GDF15 were measured from plasma samples using R&D Systems Quantikine ELISA Kits MF2100 and MGD150, respectively, according to the manufacturer’s protocols.

#### DE-2–3 cell culture and differentiation

DE-2–3 cells were cultured and differentiated essentially as described in Pan et al. (2009) and Shen et al. (2017). Briefly, cells were passaged in growth media consisting of high glucose DMEM supplemented with 10% fetal bovine serum, penicillin and streptomycin (100 U/mL), and GlutaMAX (2mM). For differentiation, cells were seeded at day −2 in differentiation medium (DM, growth media supplemented with 20nM insulin and 1nM T3) to reach confluence on day 0. On day 0, media was switched to induction media (DM supplemented with 0.5mM IBMX, 0.5 μM dexamethasone, and 0.125mM indomethacin). Media was changed to DM on day 2 and day 4. Cells were considered to be fully differentiated on day 6, and experiments were performed on day 6–9. For oxygen consumption measurements, DE-2–3 cells were differentiated in Seahorse XF24 Cell Culture Microplates, and oxygen consumption rates were measured using a Seahorse XF24 Extracellular Flux Analyzer instrument.

#### Oil Red O Stain

Oil Red O staining was performed as described in Shen et al. (2017), according to the protocol by Lonza. Briefly, 300mg of Oil Red O was dissolved in 100mL of 99% isopropanol to prepare Oil Red O stock solution. 30mL Oil Red O Stock solution was then mixed with 20mL deionized water, incubated for 10 mins at room temperature, and passed through a 0.45 mm filter to yield Oil Red O working solution. Fully differentiated DE-2–3 cells were gently rinsed with sterile DPBS and fixed for 30–60 mins in 10% formalin. The fixed cells were then rinsed with sterile water, incubated in 60% isopropanol for 2–5 mins, and incubated in Oil Red O working solution for 5 mins. The cells were rinsed with water until excess stain was removed, and then incubated for 1 min with hematoxylin counterstain. The cells were then rinsed with water until excess stain was removed, and kept under water prior to and during imaging.

### QUANTIFICATION AND STATISTICAL ANALYSIS

All statistical comparisons for low-throughput data were performed as described in the appropriate figure legends using Microsoft Excel. Significance for Student’s t tests was evaluated using a two-tailed test assuming unequal variances. Statistical analysis of the RNA-seq data was performed as described in the RNA-seq section of the STAR methods.

### DATA AND SOFTWARE AVAILABILITY

#### Software availability

Source codes for the STAR 2.4.0j (Dobin et al., 2013), HTSeq (Anders et al., 2015), and DEseq (Love et al., 2014) software packages are freely available for download through the corresponding references. GSEA and MSigDB can be freely accessed through the Broad Institute website, and is implemented via a graphical user interface. MATLAB is accessible through MathWorks, Inc. on a subscription basis.

#### Data availability

The accession number for all the sequencing results reported in this paper is GEO: GSE119964. Additional data is available by request to the lead contact.

## Supplementary Material

1

2

## Figures and Tables

**KEY RESOURCES TABLE:** 

REAGENT or RESOURCE	SOURCE	IDENTIFIER
Antibodies		
Rabbit monoclonal against MCU	Cell Signaling Technologies	Cat#14997; RRID: AB_2721812
Rabbit monoclonal against MICU1	Cell Signaling Technologies	Cat#12524; RRID: AB_2797943
Rabbit polyclonal against MICU2	Bethyl	Custom synthesized
Rabbit polyclonal against EMRE	Bethyl	Custom synthesized
Mouse monoclonal against ATP5A	Abcam	Cat#AB14748; RRID: AB_301447
Rabbit polyclonal against LRPPRC	Sigma	Cat#SAB2700419
Rabbit polyclonal against VDAC	Cell Signaling Technologies	Cat#4866; RRID: AB_2272627
Rabbit polyclonal against Actin	Cell Signaling Technologies	Cat#4967
Rabbit monoclonal against Beta-Tubulin	Cell Signaling Technologies	Cat#2128
Mouse monoclonal against PDH	Thermo Fisher	Cat#459400
Rabbit polyclonal against Phospho-PDH (S293)	EMD Millipore	Cat#ABS204
Rabbit polyclonal against Phospho-PDH (S300)	Calbiochem	Cat#AP1064
Rabbit monoclonal against ATF-4	Cell Signaling Technologies	Cat#11815; RRID: AB_2616025
Chemicals, Peptides, and Recombinant Proteins		
Phosphate buffered saline (PBS)	Thermo Fisher	Cat# 10010023
Sucrose	Sigma	Cat# S0389
HEPES	Sigma	Cat# H3375
EGTA	Sigma	Cat# E3889
KOH	Sigma	Cat# P5958
RIPA Buffer with EDTA and EGTA	Boston BioProducts	Cat# BP-115DG
Quick Start Bradford 1x Dye Reagent	Bio-Rad	Cat# 5000205
Rodent High Fat Diet	Research Diets	D12492
Trizma® hydrochloride (Tris-HCl)	Sigma	Cat# T3253
Trizma® BASE (Tris base)	Sigma	Cat# 93362
Rotenone	Sigma	Cat #R8875
Guanosine 5′-diphosphate [GDP] disodium salt	Abcam	Cat# 7415-69-2
Bovine serum albumin (fatty acid free)	Sigma	Cat# A8806
Oregon Green 488 BAPTA-6F, hexapotassium salt	Thermo Fisher	Cat# O23990
sn-Glycerol 3-phosphate bis(cyclohexylammonium) salt	Sigma	Cat# G7886
Calcium chloride	Sigma	Cat# C1016
Tetramethylrhodamine methyl ester (TMRM)	Thermo Fisher	Cat# T668
cOmplete ETDA-free protease inhibitor cocktail	Sigma	Cat# 11873580001
Protease/Phosphatase Inhibitor Cocktail	Cell Signaling Technology	Cat# 5872S
SDS sample buffer (Laemmli)	Boston BioProducts	Cat# BP-111R
QIAzol Lysis Reagent	QIAGEN	Cat# 79306
Chloroform	Sigma	Cat# 288306
QIAGEN buffer RLT	QIAGEN	Included in RNeasy Mini Kit
100% Ethanol	Decon Labs	Cat# 2716
GIBCO DMEM, high glucose	Thermo Fisher	Cat# 11965092
GIBCO Penicillin-Streptomycin (10,000 U/mL)	Thermo Fisher	Cat# 15140122
GIBCO GlutaMAX Supplement	Thermo Fisher	Cat# 35050061
Insulin	Sigma	Cat# I5500
3,3′,5-Triiodo-L-thyronine sodium salt (T3)	Sigma	Cat# T6397
Dexamethasone	Sigma	Cat# D4902
3-Isobutyl-1-methylxanthine (IBMX)	Sigma	Cat# I5879
Indomethacin	Sigma	Cat# I7378
Dulbecco’s Phosphate-Buffered Saline (DPBS)	Thermo Fisher	Cat# 14190136
Formalin solution, neutral buffered, 10%	Sigma	Cat# HT501128
Isopropanol	Sigma	Cat# I9516
Oil Red O	Sigma	Cat# O0625
Hematoxylin Solution, Harris Modified	Sigma	Cat# HHS16
Critical Commercial Assays		
RNeasy Mini Kit	QIAGEN	Cat# 74104
SuperScript® III First-Strand Synthesis SuperMix for qRT-PCR	Thermo Fisher	Cat# 11752050
TaqMan Gene Expression Master Mix	Thermo Fisher	Cat# 4369016
Mouse/Rat FGF-21 Quantikine ELISA Kit	R&D Systems	Cat# MF2100
Mouse/Rat GDF-15 Quantikine ELISA Kit	R&D Systems	Cat# MGD150
Seahorse XF24 Extracellular Flux Analyzer	Seahorse Bioscience	https://www.agilent.com/en/products/cell-analysis/seahorse-analyzers
Deposited Data		
RNA-seq results generated in this manuscript, raw and processed	NCBI Gene Expression Omnibus	http://www.ncbi.nlm.nih.gov/geo, query GEO: GSE119964
Experimental Models: Cell Lines		
DE-2-3 immortalized brown adipocytes	Gift from Dr. Bruce Spiegelman	N/A
Experimental Models: Organisms/Strains		
Mouse: C57BL/6J	The Jackson Lab	Cat# 000664
Mouse: B6FVB-Tg(UCP1-Cre)1Evdr/J	The Jackson Lab	Cat# 024670
Oligonucleotides		
MCU TaqMan Probe	Thermo Fisher	Mm01168773_m1
UCP1 TaqMan Probe	Thermo Fisher	Mm01244861_m1
PPARGC1A (PGC1A) TaqMan Probe	Thermo Fisher	Mm01208835_m1
DIO2 TaqMan Probe	Thermo Fisher	Mm00515664_m1
ATF4 TaqMan Probe	Thermo Fisher	Mm00515325_g1
ATF3 TaqMan Probe	Thermo Fisher	Mm00476033_m1
MTHFD2 TaqMan Probe	Thermo Fisher	Mm00485276_m1
CBS TaqMan Probe	Thermo Fisher	Mm00460654_m1
CTH TaqMan Probe	Thermo Fisher	Mm00461247_m1
TRIB3 TaqMan Probe	Thermo Fisher	Mm00454879_m1
VEGFA TaqMan Probe	Thermo Fisher	Mm01281449_m1
ASNS TaqMan Probe	Thermo Fisher	Mm00803785_m1
HPRT TaqMan Probe	Thermo Fisher	Mm03024075_m1
Recombinant DNA		
BAC RP23-371B1 (LB Stab)	BACPAC Resources	RP23-371B1
Software and Algorithms		
MATLAB	MathWorks	https://www.mathworks.com/products/matlab.html
STAR 2.4.0j	Picelli et al. (2014)	https://github.com/alexdobin/STAR
HTSeq	Dobin et al. (2013)	https://github.com/simon-anders/htseq
DESeq2	Anders et al. (2015)	https://bioconductor.org/packages/release/bioc/html/DESeq2.html
Gene Set Enrichment Analysis (GSEA)	Mootha et al. (2003); Subramanian et al. (2005)	http://software.broadinstitute.org/gsea/index.jsp

## Highlights

- BAT-specific MCU KO mice have normal body weight and cold tolerance
- MCU KO does not impact the gene expression response to acute cold stress in BAT
- Cold induces ATF4-dependent integrates stress response in both genotypes
- Circulating GDF15 and FGF21 rise in both genotypes in response to cold
