# Supplementary material for: Exploring the In Vivo Role of the Mitochondrial Calcium Uniporter in Brown Fat Bioenergetics
Source: Cell Rep. Author manuscript; Available in PMC 2020 May 17. (PMC7231522; doi:10.1016/j.celrep.2019.04.013)
Supplement: 1 [file NIHMS1584937-supplement-1.pdf]

Cell Reports, Volume *27*

Supplemental Information

Exploring the *In Vivo* Role of the Mitochondrial Calcium Uniporter in Brown Fat Bioenergetics

Daniel Flicker, Yasemin Sancak, Eran Mick, Olga Goldberger, and Vamsi K. Mootha

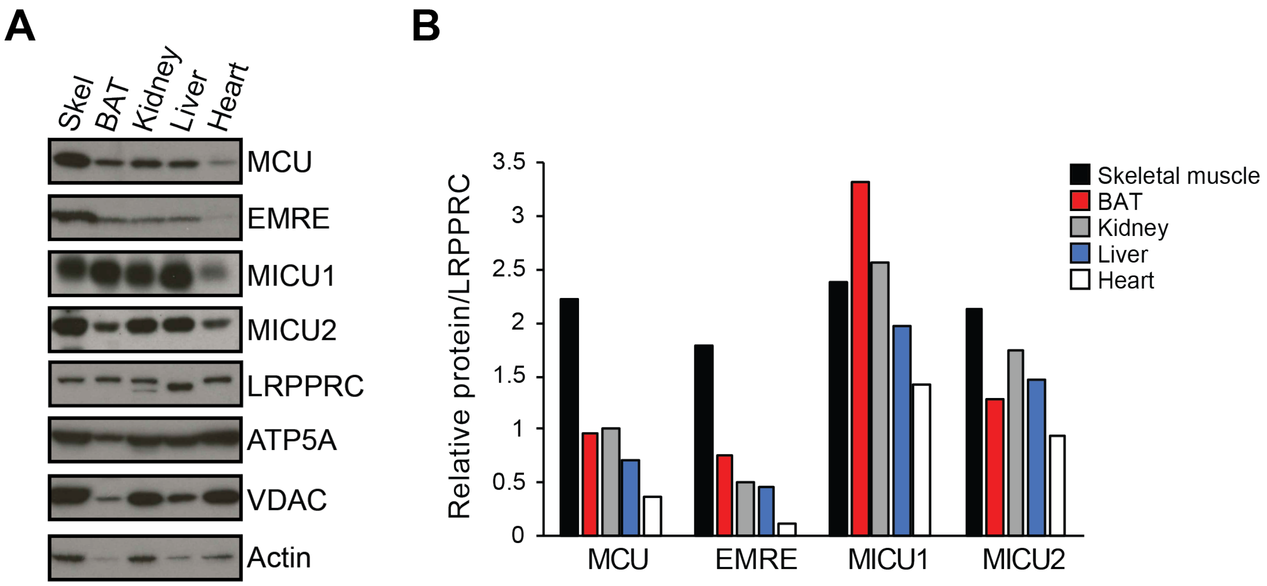

Figure S1. Tissue distribution of uniporter component proteins.
Related to Figure 1.
(A) Mitochondria were isolated from mouse skeletal muscle (gastrocnemius + soleus), BAT, kidney, liver, and heart, and lysed in RIPA buffer. Equal amounts of protein were loaded in each lane.
(B) Quantification of the uniporter component proteins in (A) relative to LRPPRC, a soluble matrix protein.

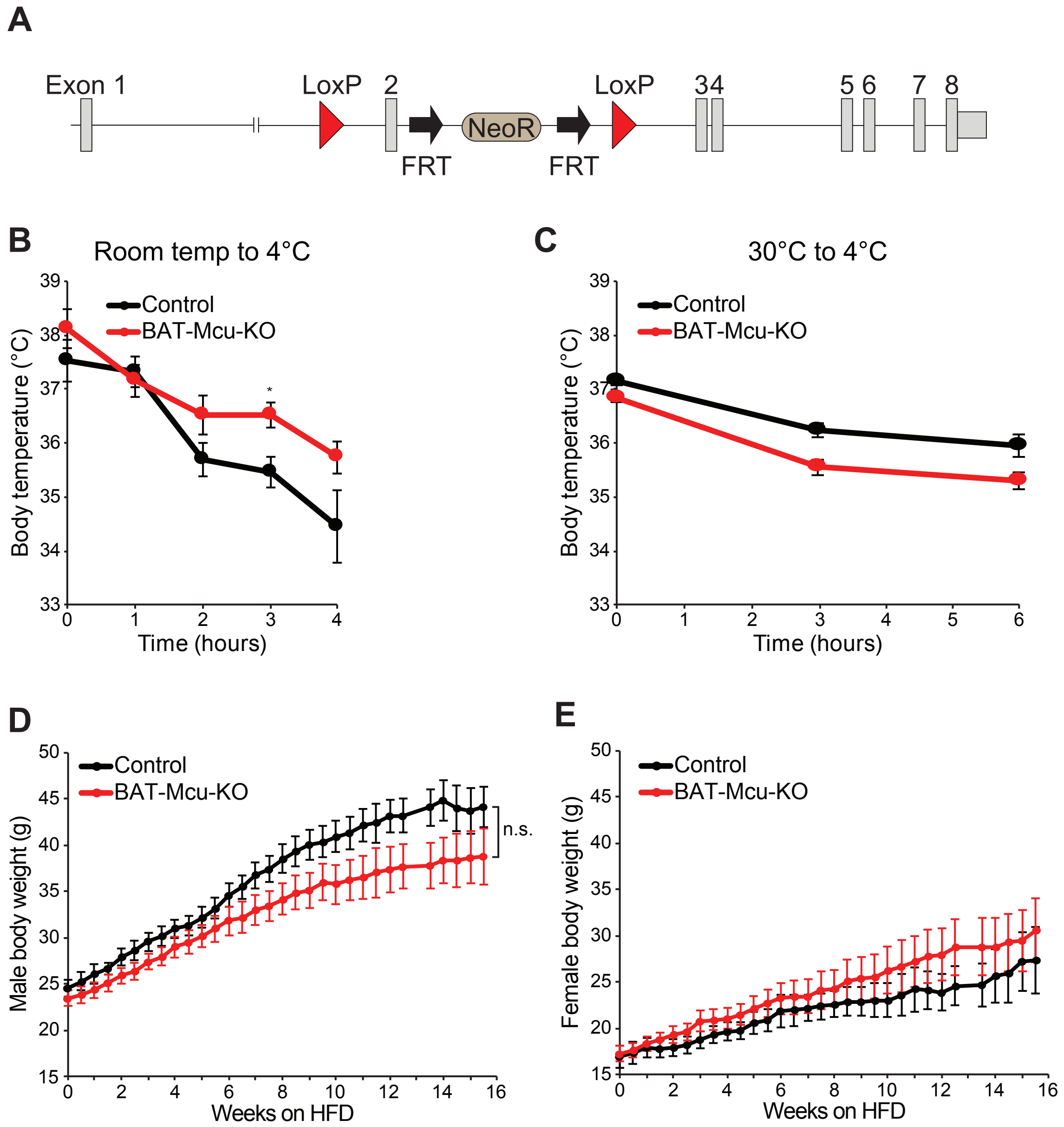

Figure S2. Construction and metabolic phenotyping of BAT-Mcu-KO mice.
Related to Figure 2.
(A) Conditional *Mcu* allele utilized to generate the *Mcu*$^{fl/fl}$ mice,
(B) Core body temperature of mice transferred from room temperature to 4°C (n = 5-6 per group).
(C) Core body temperature of mice transferred to 4°C following 1 week habituation to 30°C (n = 7 per group).
(D-E) Body mass of male (C) and female (D) -Cre and +Cre animals fed high-fat diet.

Results are reported as mean + SEM. Statistical significance is indicated as * $p < 0.05$ (student's t-test).

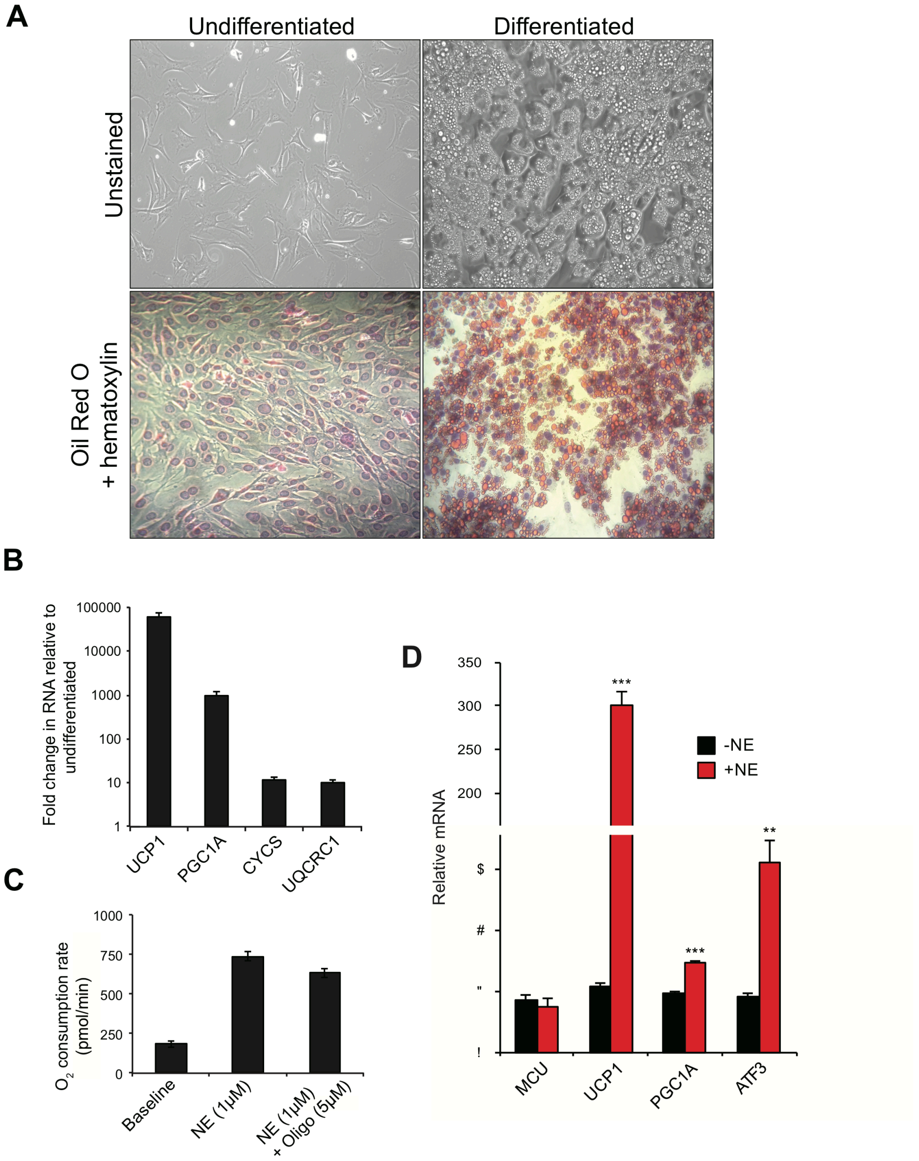

Figure S3. Transcriptional response to norepinephrine in immortalized brown adipocytes.
Related to Figure 4.
(A-C) Validation of DE-2-3 cells as an *in vitro* model of BAT physiology.
(A) Light microscopy of D-E-23 cells before and after differentiation. Oil Red O (red color) stains lipid droplets and hematoxylin (purple color) stains nuclei.
(B) Fold change of BAT marker genes in DE-2-3 cells after vs. before differentiation, as assayed by qPCR.
(C) Oxygen consumption rate in DE-2-3 cells at baseline, in response to norepinephrine (NE) treatment, and in response to NE and oligomycin (oligo) treatment.
(D) qPCR of select genes in DE-2-3 cells following stimulation with 1µM NE for 4 hours.

Results are indicated as mean + S.E.M. Statistical significance is indicated as ** $p < 0.01$, *** $p < 0.001$ (student's t-test).

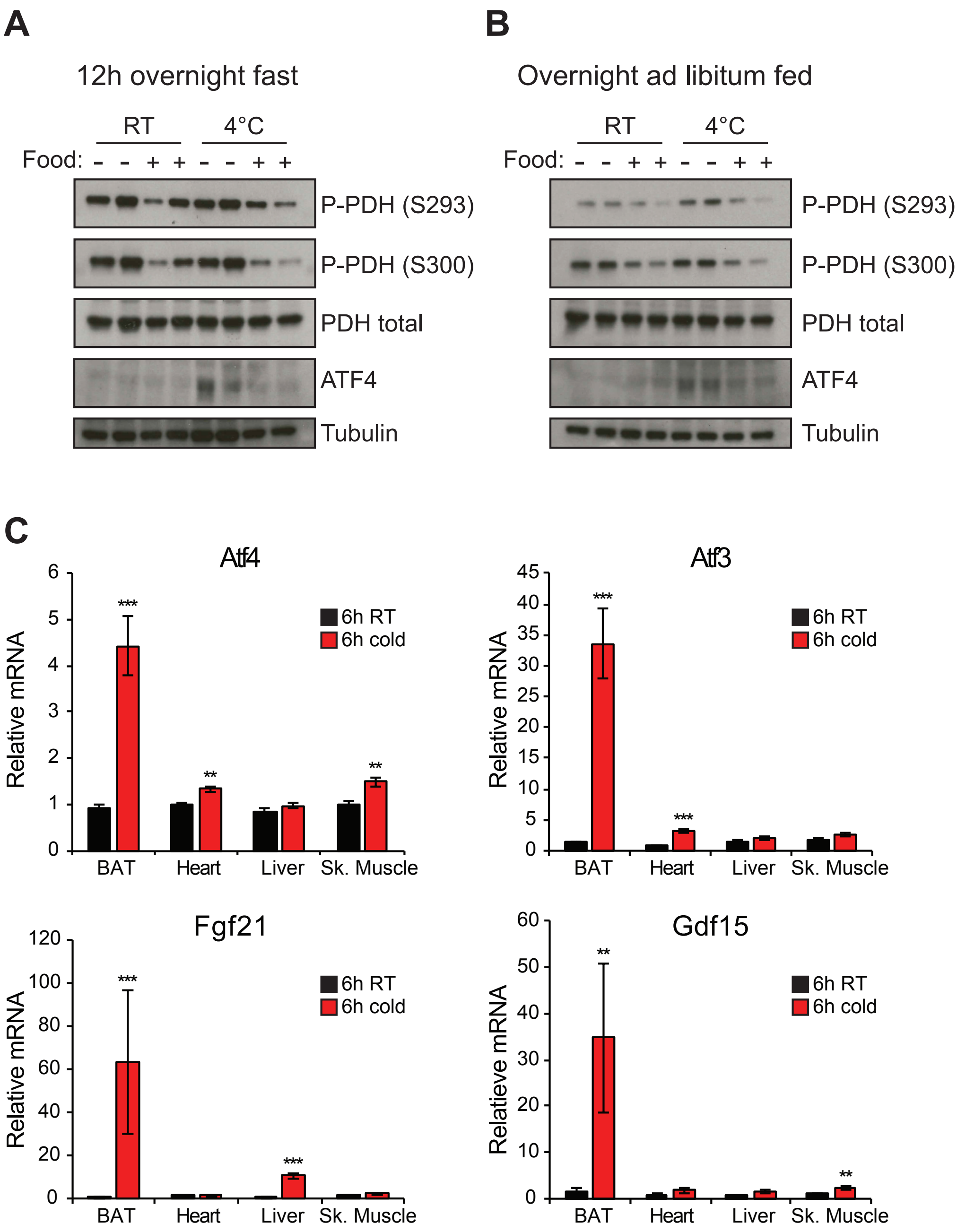

Figure S4. Cold induces the ATF4-mediated integrated stress response in BAT under fasting conditions. Related to Figure 4.

(A-B) Feeding suppresses cold-induced ATF4 accumulation. Wild-type mice were either fasted overnight (A) or provided *ad libitum* access to food (B) at room temperature (RT). Mice were then transferred to new cages at RT or 4°C, either with or without *ad libitum* access to food.

(C) Integrated stress response target gene expression in response to cold across different tissues. Wild-type C57BL6/J mice (n = 4) per group were fasted at room temperature or 4°C for 6 hours, and the indicated transcripts were quantified by qPCR.

Results are reported as mean ± SEM. Statistical significance is indicated as ** $p < 0.01$, *** $p < 0.001$ (student's t-test).
